# PD-1 and Tim-3 pathways are associated with regulatory CD8^+^ T-cell function in decidua and maintenance of normal pregnancy

**DOI:** 10.1038/cddis.2015.112

**Published:** 2015-05-07

**Authors:** S-C Wang, Y-H Li, H-L Piao, X-W Hong, D Zhang, Y-Y Xu, Y Tao, Y Wang, M-M Yuan, D-J Li, M-R Du

**Affiliations:** 1 Laboratory for Reproductive Immunology, Hospital and Institute of Obstetrics and Gynecology, Fudan University Shanghai Medical College, Shanghai 200011, China; 2 Shanghai Key Laboratory of Female Reproductive Endocrine Related Diseases, Shanghai 200011, China; 3 Department of Immunology, School of Basic Medical Sciences, Fudan University, Shanghai, China

## Abstract

CD8^+^ T cells are critical in the balance between fetal tolerance and antiviral immunity. T-cell immunoglobulin mucin-3 (Tim-3) and programmed cell death-1 (PD-1) are important negative immune regulatory molecules involved in viral persistence and tumor metastasis. Here, we demonstrate that Tim-3^+^PD-1^+^CD8^+^ T cells from decidua greatly outnumbered those from peripheral blood during human early pregnancy. Co-culture of trophoblasts with CD8^+^ T cells upregulated PD-1^+^ and/or Tim-3^+^ immune cells. Furthermore, the population of CD8^+^ T cells co-expressing PD-1 and Tim-3 was enriched within the intermediate memory subset in decidua. This population exhibited high proliferative activity and Th2-type cytokine producing capacity. Blockade of Tim-3 and PD-1 resulted in decreased *in vitro* proliferation and Th2-type cytokine production while increased trophoblast killing and IFN-*γ* producing capacities of CD8^+^ T cells. Pregnant CBA/J females challenged with Tim-3 and/or PD-1 blocking antibodies were more susceptible to fetal loss, which was associated with CD8^+^ T-cell dysfunction. Importantly, the number and function of Tim-3^+^PD-1^+^CD8^+^ T cells in decidua were significantly impaired in miscarriage. These findings underline the important roles of Tim-3 and PD-1 pathways in regulating decidual CD8^+^ T-cell function and maintaining normal pregnancy.

Successful pregnancy requires the maternal immune system to tolerate the semi-allogeneic fetus. A failure in immune tolerance may result in abnormal pregnancies, such as recurrent spontaneous abortion. For many years, the model of immune regulation during pregnancy has been based on a shift in the maternal immune response towards a Th2 bias. The shift from producing inflammatory Th1-type cytokines toward Th2-type cytokines promotes maternal–fetal tolerance.^1, 2^ In addition, maternal administration of the Th2-type cytokine interleukin (IL)-10 or blockade of the Th1-type cytokine tumor necrosis factor (TNF)-*α* is known to prevent pregnancy loss induced by lipopolysaccharide.^3, 4^

Compared with CD4^+^ T cells, our understanding of the role of CD8^+^ T cells during pregnancy remains poorly understood. CD8^+^ T cells, which directly recognize allogeneic major histocompatibility complex (MHC) class I molecules, have important roles in defense against viral infections. Studies on several murine models have demonstrated the existence of CD8^+^ T cells at the maternal–fetal interface.^5^ During normal pregnancy, the major antigen present is the embryo-derived paternal antigen expressed on extravillous trophoblast (EVT) cells. These cells do not express MHC class I human leukocyte antigens (HLA)-A and HLA-B,^6^ which are the main causes of CD8^+^ T cell-mediated rejection. However, HLA-C and HLA-G, highly expressed on EVT cells,^6^ can elicit a direct cytotoxic response by CD8^+^ T cells during hematopoietic stem cell and allogeneic organ transplantation.^7, 8^ Therefore, whether suppressor or regulatory CD8^+^ T cells are present at the maternal–fetal interface, and how they function to maintain normal pregnancy, remain to be explored.

Inhibitory co-stimulatory signals have crucial roles in regulating CD8^+^ T-cell activation or tolerance. It has been shown that exhausted T cells express up to seven different inhibitory molecules,^9^ including PD-1 and Tim-3. PD-1 has been identified as a marker for dysfunctional T cells, and blockade of PD-1 signals has been shown to revert the dysfunctional state of exhausted CD8^+^ T cells in most cases.^10, 11^ Tim-3 has been similarly associated with CD8^+^ T-cell exhaustion as Tim-3 blockade restores proliferation and cytokine production.^12, 13^ Tim-3 and PD-1 co-expression on T cells characterizes the most severely exhausted CD8^+^ T-cell subset, and combined blockade of Tim-3 and PD-1 restores the function of exhausted CD8^+^ T cells.^14, 15, 16^ However, much less is known about the functional regulation of Tim-3 and PD-1 on CD8^+^ T cells during pregnancy.

In this study, we investigated Tim-3 and PD-1 expression on CD8^+^ T cells from decidua and peripheral blood in normal pregnant women and those who underwent miscarriage. In particular, we used surface and intracellular phenotype analysis, as well as multifunctional assays, to study the role of Tim-3 and PD-1 signaling pathways in regulating decidual CD8^+^ (dCD8^+^) T-cell function and maintenance of pregnancy. Our data indicate that Tim-3 and PD-1 co-expression on CD8^+^ T cells might be important in maintaining maternal–fetal immune tolerance and successful pregnancy. These results could provide a strategy for developing novel therapies that enhance Tim-3 and PD-1 signals to promote maternal–fetal tolerance and prevent pregnancy loss.

## Results

### Tim-3 and PD-1 co-expression on CD8^+^ T cells in early pregnancy

**Figure 1:**
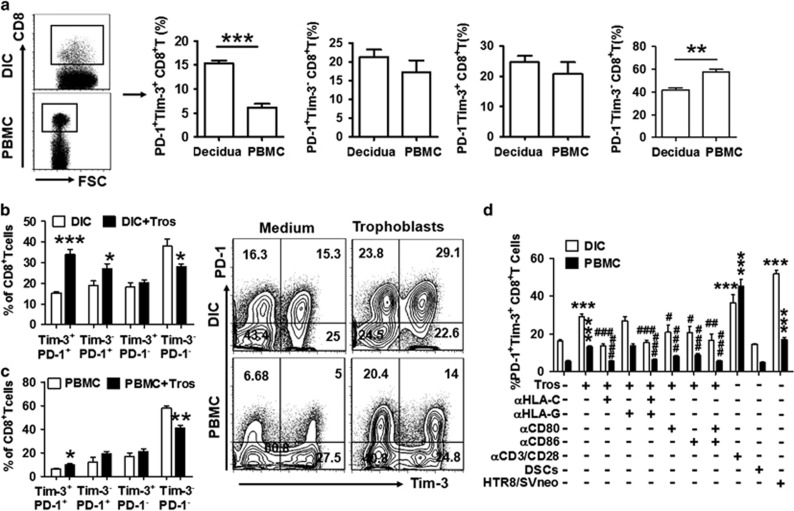
PD-1 and Tim-3 expression on CD8^+^ T cells during pregnancy. (**a**) Frequency of Tim-3 and PD-1 expression on gated CD8^+^ T cells from peripheral blood mononuclear cells (PBMCs) and decidual immune cells (DICs) during human first trimester pregnancy. Freshly isolated PBMCs and DICs were stained with antibodies against CD8, PD-1, and Tim-3 to assess PD-1 and Tim-3 expression on CD8^+^ T cells by flow cytometry. *n*=30. **P<0.01; ***P<0.001. (**b** and **c**) Freshly isolated trophoblasts were seeded at 2 × 10^5^ cells/ml per well in matrigel-coated 24-well plates overnight. The supernatants were aspirated completely, and the cells were washed twice with phosphate-buffered saline (PBS). Equal numbers of DICs or PBMCs were added to each well. Flow-cytometric analysis (right) and quantification (left) of Tim-3 and PD-1 expression on decidual (**b)** and peripheral (**c**) CD8^+^ T cells with or without co-culture with trophoblasts (Tros) for 48 h. *n*=9. *P<0.05; **P<0.01; ***P<0.001. (**d**) Freshly isolated trophoblasts (Tros), decidual stromal cells (DSCs), and human HTR8/SVneo cells were seeded at 2 × 10^5^ cells/ml per well in 24-well plates. After overnight, the supernatants were aspirated completely, and the cells were washed twice with PBS. Equal numbers of DICs or PBMCs were added to each well. The anti-CD3 and anti-CD28 antibodies were used to activate CD8^+^ T cells in some wells. ***P<0.001, compared with the control. ^#^*P*<0.5, ^##^*P*<0.01, ^###^*P*<0.001, compared with the group co-cultured with trophoblasts. Data represent mean±S.E.M. The flow-cytometry plots are representative of three independent experiments. *α*HLA-C: anti-HLA-C antibody, *α*HLA-G: anti-HLA-G antibody, *α*CD80: anti-CD80 antibody, *α*CD86: anti-CD86 antibody, *α*CD3/CD28: anti-CD3 antibody plus anti-CD28 antibody

To investigate the potential role of Tim-3 and PD-1 in CD8^+^ T-cell function during pregnancy, we first examined their expressions on CD8^+^ T cells and found that cells co-expressing Tim-3 and PD-1 comprise about 15% of dCD8^+^ T cells and less than 6% of peripheral CD8^+^ (pCD8^+^) T cells in early pregnancy (Figure 1a). In contrast, Tim-3^−^PD-1^−^CD8^+^ T cells accounted for over 55% of PBMCs and around 40% of decidual immune cells (DICs). These results demonstrate that Tim-3^+^PD-1^+^CD8^+^ T cells are preferentially distributed in decidua.

Previously, we have shown that embryonic trophoblasts have the unique ability to instruct DICs.^17, 18^ Then, we determined whether trophoblasts could contribute to the higher expression of Tim-3 and PD-1 on CD8^+^ T cells. The results showed that co-culture of trophoblast cells increased the frequency of Tim-3^+^PD-1^+^CD8^+^ T cells and decreased Tim-3^−^PD-1^−^CD8^+^ T cells in both DICs and PBMCs (Figures 1b and c). These data indicate that embryonic trophoblast cells carrying paternal antigens can upregulate Tim-3 and PD-1 expression on CD8^+^ T cells, resulting in a higher percentage of CD8^+^ T cells co-expressing Tim-3 and PD-1 in decidua.

To further explore the mechanisms underlying the upregulation of Tim-3 and PD-1 expression on CD8^+^ T cells, we co-cultured DICs or PBMCs with different control cell populations including HTR8/SVneo, an immortalized human extra-villious trophoblast cell line, and decidual stromal cells (DSC, maternal-derived cells at the maternal–fetal interface). We also directly activated CD8^+^ T cells using anti-CD3/CD28 antibodies. The results in Figure 1d showed that primary trophoblasts and HTR-8/SVneo cells, but not DSC cells, upregulated the percentage of PD-1^+^Tim-3^+^CD8^+^ T cells in PBMCs and DICs, suggesting that embryo-derived trophoblasts promoted Tim-3 and PD-1 expression on CD8^+^ T cells. The percentage of CD8^+^ T cells co-expressing Tim-3 and PD-1 also increased after activation of CD8^+^ T cells by anti-CD3/CD28 antibodies. Furthermore, blockade of CD80/CD86 signals inhibited trophoblast-induced Tim-3 and PD-1 upregulation on CD8^+^ T cells. These data indicate that embryonic trophoblasts might upregulate Tim-3 and PD-1 co-expression on CD8^+^ T cells at least partially through activation of these cells. To elucidate the mechanism by which trophoblasts induced the higher percentage of Tim-3^+^PD-1^+^CD8^+^ T cell at the maternal–fetal interface, HLA-G and HLA-C blocking antibodies were used since these trophoblast-expressing molecules are critical for embryo-induced maternal–fetal immune tolerance. Interestingly, administration of anti-HLA-C, but not anti-HLA-G, antibody significantly inhibited trophoblast-induced upregulation of Tim-3 and PD-1 co-expression on CD8^+^ T cells. These data suggest that the higher percentage of PD-1^+^Tim-3^+^CD8^+^ T cells at the maternal–fetal interface is promoted by embryonic trophoblasts in an HLA-C-restricted manner.

In addition, we studied the effect of soluble Th1 and Th2 cytokines (IL-4 and IFN-*γ*), as well as PD-1 and Tim-3 ligands (PD-L1 and galectin-9, respectively) expressed by trophoblasts (data not shown), on Tim-3 and PD-1 co-expression on CD8^+^ T cells. As shown in Supplementary Figure S1A and B, none of these factors was involved in regulating Tim-3 and PD-1 expression by trophoblasts. These data were confirmed by the fact that trophoblasts did not affect Tim-3 and PD-1 co-expression on CD8^+^ T cells after separation of trophoblasts and CD8^+^ T cells with a transwell insert in the co-culture system.

### Function of dCD8^+^ T cells expressing Tim-3 and PD-1 during normal pregnancy

**Figure 2:**
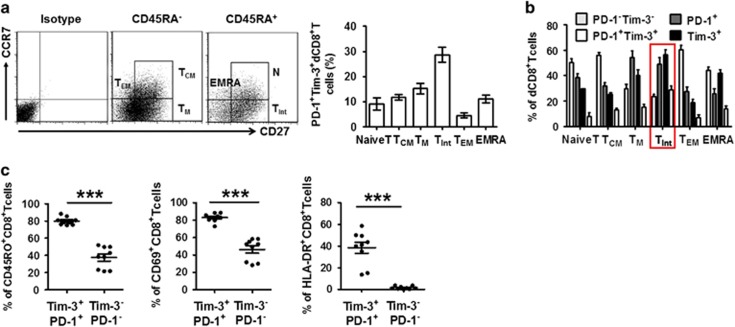
Decidual CD8^+^ T cells co-expressing Tim-3 and PD-1 display a T_Int_ phenotype. (**a** and **b**) Decidual CD8^+^ T (dCD8^+^ T) cells from first trimester pregnancy (*n*=12) were stained with antibodies against CD45RA, CCR7, CD27, or their respective isotype controls to determine their differentiation phenotype (see Results). (**a**) Frequency of PD-1^+^Tim-3^+^ cells within each population. Cells expressing both PD-1 and Tim-3 were predominantly of the T_Int_ phenotype. Data represent mean±S.E.M. The flow-cytometry plot is representative of four independent experiments. (**b**) Frequency of PD-1^–^Tim-3^–^, PD-1^+^, Tim-3^+^, and PD-1^+^Tim-3^+^ cells within each population. (**c**) Decidual CD8^+^ T cells from first trimester pregnancy were stained with antibodies against PD-1, Tim-3, CD69, HLA-DR, and CD45RO. The PD-1^+^Tim-3^+^ and PD-1^–^Tim3^–^ phenotypes were compared. *n*=9. Data represent mean ±S.E.M. from three independent experiments. A significant portion of PD-1^+^Tim-3^+^ T cells expressed all three activation/memory markers. ***P<0.001

To further characterize the different subsets of dCD8^+^ T cells, we first examined the expression of CD45RA, CD27, and CCR7 on these cells. The differential expression of these three molecules was conventionally thought to define naïve and memory T cells. Based on these markers, T cells are classified as naïve T (CD27^+^CD45RA^+^CCR7^+^), T_CM_ (CD45RA^−^CD27^+^CCR7^+^), T_M_ (CD45RA^−^CD27^+^CCR7^−^), T_Int_ (CD45RA^+^CD27^dim^CCR7^−^), T_EM_ (CD45RA^−^CCR7^−^CD27^−^), and EMRA (CD45RA^+^CD27^−^CCR7^−^) cells.^16, 19^ Within these subsets, the vast majority of the population expressed lower amounts of either Tim-3 or PD-1 (Figure 2a). However, within the T_Int_ subset of dCD8^+^ T cells, a higher proportion demonstrated dual or single expression of PD-1 and Tim-3 (Figure 2b). We next examined the expression of CD69, HLA-DR, and CD45RO to determine whether T cell pools varied with regard to Tim-3 and PD-1 expression during pregnancy. We observed that PD-1^+^Tim-3^+^CD8^+^ T cells expressed higher levels of activation (CD69 and HLA-DR) and memory (CD45RO) markers than did PD-1^−^Tim-3^−^T cells (Figure 2c).

**Figure 3:**
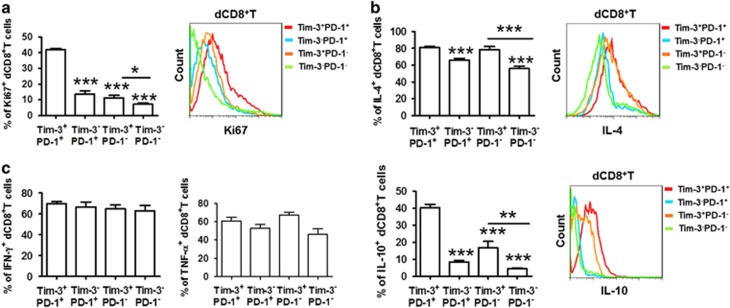
Proliferation and cytokine production in dCD8^+^ T cells during normal pregnancy. (**a**) Freshly isolated DICs were stained with antibodies against Ki-67 to detect the proliferation of dCD8^+^ T cells by flow cytometry. A representative flow-cytometry plot (right) and quantification (left) of Ki-67 staining in dCD8^+^ T cells are shown. *n*=9. (**b** and **c**) Freshly isolated DICs were treated with brefeldin A (10 *μ*g/ml), phorbol 12-myrstate 13-acetate (PMA) (50 ng/ml), and ionomycin (1 *μ*g/ml) for 4 h, then the cells were harvested and analyzed by flow cytometry. (**b**) Expression of the Th2-type cytokines IL-4 and IL-10 in Tim-3^+^PD-1^+^, Tim-3^−^PD-1^+^, Tim-3^+^PD-1^−^, and Tim-3^−^PD-1^−^ dCD8^+^ T cells. A representative flow-cytometry plot (right) and quantitation (left) are shown. *n*=9. (**c**) Quantitation of flow-cytometric analysis of the Th1-type cytokines IFN-*γ* and TNF-*α* in Tim-3^+^PD-1^+^, Tim-3^−^PD-1^+^, Tim-3^+^PD-1^−^, and Tim-3^−^PD-1^−^ dCD8^+^ T cells. *n*=12. Data represent mean±S.E.M. The flow-cytometry plots are representative of three independent experiments. *P<0.05, ***P<0.001, compared with Tim-3^+^PD-1^+^ dCD8^+^ T cells

Ki-67 is generally used as a marker of proliferating cells.^20^ As seen in Supplementary Figure S2 and Figure 3a, the majority of the expanded population of T cells was Tim-3^+^PD-1^+^, whereas proliferating Tim-3^−^PD-1^−^CD8^+^ T cells were restricted both at the maternal–fetal interface and in PBMCs. We also measured CD107a^21^ expression to assess the cytolytic activity of dCD8^+^ T cells with varying expression profiles of Tim-3 and PD-1. Unexpectedly, CD107a expression did not vary in the different CD8^+^ T-cell subsets (Supplementary Figure S3A).

To determine whether Tim-3- and/or PD-1-expressing dCD8^+^ T cells exhibited a tolerant phenotype, we examined the production of Th1-type cytokines (IFN-*γ* and TNF-*α*), the Th2-type cytokine IL-4, and the regulatory cytokine IL-10. We found that PD-1^+^Tim-3^+^CD8^+^ T cells were associated with increased Th2-type cytokine expression (Figure 3b). However, it appears that there is no difference in Th1-type cytokine (Figure 3c) or another regulatory cytokine TGF-*β*1 (Supplementary Figure S3B) production by CD8^+^ T cells expressing Tim-3 and PD-1 alone, both or neither. These data indicate that Tim-3^+^PD-1^+^CD8^+^ T cells display higher proliferative activity and produce more Th2-type cytokines than Tim-3^−^PD-1^−^CD8^+^ T cells.

### Effects of targeting Tim-3 and PD-1 on dCD8^+^ T cells

**Figure 4:**
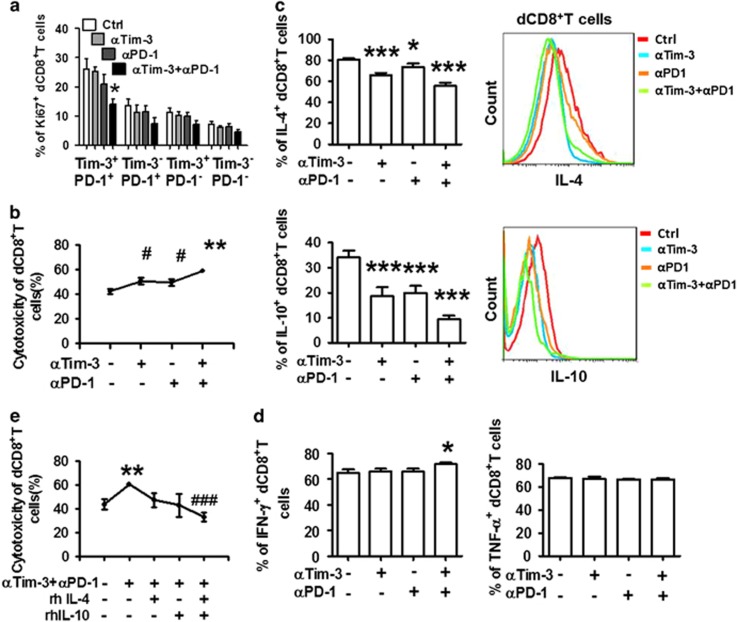
Effect of blocking Tim-3 and PD-1 signaling pathways on dCD8^+^ T-cell function. (**a**) Quantitation of flow-cytometric analysis of Ki-67 staining on different subsets of dCD8^+^ T cells cultured for 48 h in the presence or absence of anti-Tim-3 antibody (10 *μ*g/ml), anti-PD-1 antibody (10 *μ*g/ml), or both. *n*=6. (**b**) Cytotoxic activity of dCD8^+^ T cells (effector) following treatment with the indicated blocking antibodies (as above) on trophoblasts (target cells). The cytotoxic activities were measured at an effector to target (E : T) ratio of 100 : 1. The percentage of cytotoxicity was calculated after correcting for lactose dehydrogenase (LDH) release from Tros using the formula: Percent cytotoxicity=100 × (LDH_Experimental_−LDH_Culture Medium Background_)/(LDH_Maximal_−LDH_Culture Medium Background_). LDH_experimental_ represents LDH release activity from co-cultures of effector and target cells. LDH_Culture Medium Background_ represents LDH release from Tros. LDH_maximal_ represents LDH release from Tros that were lysed by sonication. **P<0.01, compared with the control group. ^#^*P*<0.01, compared with the group treated with anti-Tim-3 plus anti-PD-1 antibodies. *n*=12. (**c** and **d**) Quantification (left) and representative flow-cytometric plots (right) of cytokine production by dCD8^+^ T cells following treatment with the indicated blocking antibodies. Brefeldin A (10 *μ*g/ml), PMA (50 ng/ml), and ionomycin (1 *μ*g/ml) were added 4 h before the end of the 48-h culture, then the cells were harvested and analyzed by flow cytometry. (**e**) Cytotoxic activity of dCD8^+^ T cells (effector) following different treatments (as above) on trophoblasts (target cells). Decidual CD8^+^ T cells (effector cells; 100 *μ*l) at concentrations of 2.0 × 10^6^ cells/ml were treated with anti-Tim-3 plus anti-PD-1 (10 *μ*g/ml), anti-Tim-3 plus anti-PD-1 plus IL-4 (10 ng/ml), anti-Tim-3 plus anti-PD-1 plus IL-4 (10 ng/ml), anti-Tim-3 plus anti-PD-1 plus IL-4 plus IL-10 for 48 h, and then mixed with trophoblasts at a ratio of 100 : 1 in a 96-well plate. Data represent the mean±S.E.M. *n*=12. *P<0.05, **P<0.01, ***P<0.001, compared with the control group. ^###^*P*<0.001, compared with the group treated with anti-Tim-3 plus anti-PD-1 antibodies. *n*=6. *α*Tim-3: anti-Tim-3 antibody; *α*PD-1: anti-PD-1 antibody; rhIL-4: recombinant human IL-4; rhIL-10: recombinant human IL-10

As noted previously, separate or combined blockade of Tim-3 and PD-1 can restore the function of exhausted CD8^+^ T cells.^10, 12, 14^ We explored the effects of single or dual PD-1 and Tim-3 blockade on dCD8^+^ T-cell proliferation and cytotoxic activity. Unlike the results observed in infection and cancer, Tim-3 or PD-1 blockade displayed a trend, but not a significant decrease, in proliferation as assessed by Ki-67 expression (Figure 4a). Combined blockade of the signaling pathways resulted in reduced Ki-67 expression. This effect was only observed in Tim-3^+^PD-1^+^CD8^+^ T cells, indicating the important role of Tim-3 and PD-1 in the enrichment of decidual regulatory CD8^+^ T cells.

Because trophoblasts express paternal antigen, and therefore may be eliminated by dCD8^+^ T cells, we examined the cytolytic activity of dCD8^+^ T cells toward trophoblasts using the lactate dehydrogenase (LDH) assay. Decidual CD8^+^ T cells induced cell lysis of target trophoblast cells in a dose-dependent manner (Supplementary Figure S4). We next evaluated whether Tim-3 and PD-1 signaling pathways are involved in regulating dCD8^+^ T-cell cytolytic activity. Tim-3 blockade increased the killing capacity of dCD8^+^ T cells at levels comparable to PD-1 blockade. Furthermore, dual blockade of Tim-3 and PD-1 pathways produced an additive effect, leading to a maximal boost in cytolysis (Figure 4b).

To further address the effect of Tim-3 and PD-1 antibody treatment on dCD8^+^ T-cell function and the immune microenvironment at the maternal–fetal interface, we isolated whole DICs from normal pregnancy and cultured them with the antibodies as described earlier. We found that treatment with anti-Tim-3 or anti-PD-1 antibody alone significantly decreased IL-4 and IL-10 production in dCD8^+^ T cells. This decrease was especially notable in the dual blockade of Tim-3 and PD-1 signals (Figure 4c). We also observed a similar trend in the production of IL-5, another Th2-type cytokine, but not TGF-*β* (Supplementary Figure S5). Although we observed little increase in IFN-*γ* production after combined Tim-3 and PD-1 blockade, Th1-type cytokines possessed weak reactivity to Tim-3 and PD-1 blockade (Figure 4d). In addition, we observed that there was no difference in CD8^+^ T-cell viability following treatment with the antibodies (Supplementary Figure S5B).

Because PD-1 and Tim-3 blockade increased dCD8^+^ T-cell cytotoxicity and decreased IL-4 and IL-10 production, we postulated that these cytokines are responsible for the inhibition of cytotoxicity. Administration of human recombinant IL-4 or IL-10 decreased the killing capacity of dCD8^+^ T cells in a dose-dependent manner (Supplementary Figure S5C). Combined administration of IL-4 and IL-10 enhanced this effect. Furthermore, we observed that pretreatment with IL-4 and IL-10 completely reversed the effect of anti-Tim-3 and anti-PD-1 antibodies on inducing dCD8^+^ T-cell cytotoxicity (Figure 4e).

### *In vivo* roles of Tim-3 and PD-1 during early pregnancy

**Figure 5:**
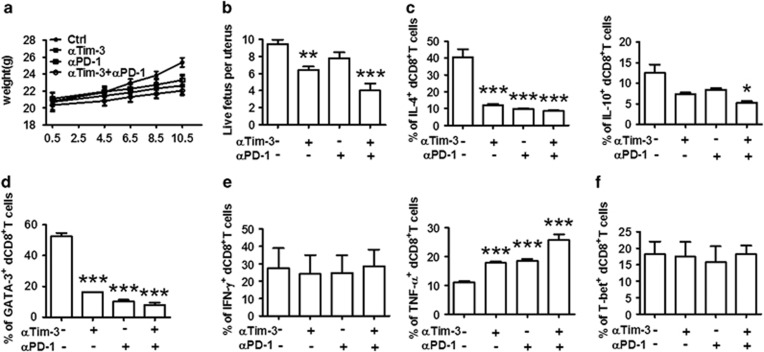
*In vivo* roles of Tim-3 and PD-1 during early pregnancy. (**a**) The weight of pregnant CBA/J females treated with PBS, anti-Tim-3 antibody, anti-PD-1 antibody, or both antibodies i.p. at doses of 500, 250, and 250 mg at days 4.5, 6.5, and 8.5, respectively. (**b**) The number of live fetuses per uterus from pregnant CBA/J females following treatment with the indicated blocking antibodies. Pregnant mice were kiled at day 10.5 of pregnancy, the uteri were removed, and the implantation sites and resorbed/live embryos were counted to assess the effect of anti-Tim-3 and anti-PD-1 antibody treatment on pregnancy. (**c**–**f**) Quantification of flow-cytometric analysis of cytokine production and transcription factor expression by dCD8^+^ T cells of mice following treatment with the indicated blocking antibodies. Freshly isolated DICs were treated with brefeldin A (10 *μ*g/ml), PMA (50 ng/ml), and ionomycin (1 *μ*g/ml) for 4 h, then the cells were harvested and analyzed by flow cytometry. Data represent mean±S.E.M. of *n*=6–8 mice per group and are representative of four independent analyses. *P<0.05, **P<0.01, ***P<0.001, compared with the control group. *α*Tim-3: anti-Tim-3 antibody; *α*PD-1: anti-PD-1 antibody

To further investigate the role of Tim-3 and PD-1 in maternal–fetal tolerance and normal pregnancy maintenance, we established a mouse pregnancy model by mating BALB/c males with CBA/J females. Then, we challenged the pregnant CBA/J females with Tim-3 and/or PD-1 blocking antibodies. Treatment with either blocking antibody caused decreased growth in body weight (Figure 5a). The antibody-treated mice were more susceptible to fetal loss as manifested by a reduction in the number of live fetuses per uterus (Figure 5b). Furthermore, dual blockade of Tim-3 and PD-1 pathways had a combined effect, leading to the least body weight growth and live fetus number per uterus (Figures 5a and b). These data indicate that Tim-3 and PD-1 could have a protective role during successful pregnancy *in vivo*.

Next, we tested whether fetal loss following *in vivo* blockade resulted from CD8^+^ T-cell dysfunction. As expected, the production of Th2-type cytokines by CD8^+^ T cells decreased following anti-Tim-3 or anti-PD-1 antibody challenge. Combined blockade further reduced Th2-type cytokine production compared with single antibody blockade (Figure 5c). To validate the decrease in Th2-type cytokines by dCD8^+^ T cells, we examined the expression of a Th2-type master transcription factor GATA-3.^22^ Our data demonstrated that GATA-3 expression was greatly reduced in dCD8^+^ T cells following single or combined antibody blockade (Figure 5d). In addition, treatment with anti-Tim-3 and/or anti-PD-1 antibody did not affect the expression of IFN-*γ* or T-bet, the Th1 master regulator (Figure 5e).^22^ However, TNF-*α* production by CD8^+^ T cells was upregulated. Similar phenomena were observed in splenocytes (Supplementary Figure S6). Taken together with our *in vitro* data, Th2 but not Th1 immune responses function alongside Tim-3 and PD-1 during pregnancy.

### Decreased number of Tim-3^+^PD-1^+^CD8^+^ T cells with deficient function in miscarriage

**Figure 6:**
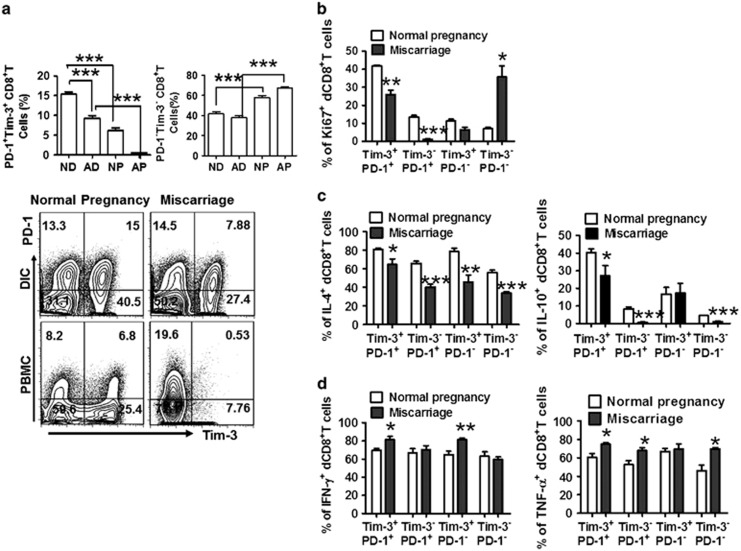
Decreased number of CD8^+^ T cells co-expressing Tim-3 and PD-1 with disregulated cytokine production is observed in human early pregnancy loss. (**a**) Frequency of Tim-3 and PD-1 expression on gated CD8^+^ T cells from DICs and PBMCs from normal pregnancy (NP; *n*=30) and miscarriage (abnormal pregnancy, AP; *n*=36) as measured by flow cytometry. (**b**) Flow-cytometric analysis of Ki-67 staining in dCD8^+^ T cells from normal pregnancy (*n*=30) and miscarriage (*n*=36). (**c** and **d**) Quantification of flow-cytometric analysis of cytokine production by dCD8^+^ T cells from normal pregnancy (*n*=30) and miscarriage (*n*=36). Freshly isolated DICs were treated with brefeldin A (10 *μ*g/ml), PMA (50 ng/ml), and ionomycin (1 *μ*g/ml) for 4 h, then the cells were harvested, fixed, permeabilized, and stained with antibodies against IL-4, IL-10, IFN-*γ*, TNF-*α*, CD8, PD-1, and Tim-3. Data represent mean±S.E.M. The flow-cytometry plot is representative of six independent experiments. *P<0.05, **P<0.01, ***P<0.001

The co-expression of Tim-3 and PD-1 on CD8^+^ T cells is conducive to the decreased cytolytic activity of CD8^+^ T cells and the development of a Th2-dominant milieu in the decidua, which is essential for maintaining normal pregnancy. Therefore, we investigated whether decreased numbers and/or defective functionality of Tim-3^+^PD-1^+^CD8^+^ T cells can be observed in miscarriage patients. As expected, compared with normal pregnancy, the dual expression of Tim-3 and PD-1 on dCD8^+^ T cells was significantly reduced in miscarriage (Figure 6a). Although the number of Tim-3^−^PD-1^−^CD8^+^ T cells was stable in the decidua from miscarriage, these cells expressed significantly higher levels of Ki-67 (Figure 6b). In contrast, decidual Tim-3^+^PD-1^+^CD8^+^ T cells expressed lower levels of Ki-67 (Figure 6b), suggesting that the immune regulatory Tim-3^+^PD-1^+^CD8^+^ T cells were less proliferative during miscarriage. Moreover, in normal pregnancy, Tim-3^+^PD-1^+^ CD8^+^ T cells, the number of which was more than that in miscarriage, produced higher levels of IL-4 and IL-10 but lower levels of IFN-*γ* and TNF-*α*. In parallel, Tim-3^−^PD-1^−^ dCD8^+^ T cells in miscarriage produced lower amounts of Th2-type cytokines, but more TNF-*α* (Figures 6c and d).

## Discussion

During early gestation, EVT cells expressing paternal antigen penetrate deeply into the decidua^6^ and can elicit a direct cytotoxic response by CD8^+^ T cells.^7, 8^ Regulatory or suppressor CD8^+^ T cells have been proposed to contribute to fetal tolerance.^23, 24, 25^ In this study, we verified the co-expression of Tim-3 and PD-1 on CD8^+^ T cells during early pregnancy. Interestingly, the dual expression of Tim-3 and PD-1 on dCD8^+^ T cells from normal pregnancy was much higher than that on pCD8^+^ T cells while Tim-3^−^PD-1^−^CD8^+^ T cells were less abundant in the decidua. Embryonic trophoblasts contributed to the increased expression of Tim-3 and PD-1 on CD8^+^ T cells in an HLA-C-restricted manner. These results provide further evidence that maternal immune cells could be educated by embryonic trophoblasts to develop a unique phenotype and maintain fetal tolerance.^17, 18, 26, 27^ Importantly, the number and function of Tim-3^+^PD-1^+^CD8^+^ T cells in decidua were significantly impaired in miscarriage, suggesting that the expression of inhibitory molecules Tim-3 and PD-1 on CD8^+^ T cells during pregnancy might conduce to the maintenance of maternal–fetal immune tolerance and normal pregnancy.

Tim-3 and PD-1 are well-known inhibitory co-stimulatory signals that contribute to the exhaustion of T cells.^9^ Tim-3^+^PD-1^+^CD8^+^ T cells are highly dysfunctional with markedly reduced cytotoxic activity and cytokine production.^15, 28^ Based on their predicted ability to survive and proliferate, CD8^+^ T sub-populations can be ranked (from highest to lowest) as follows: Naive→T_CM_→T_M_→T_Int_→T_EM_→EMRA.^19^ Within the T_Int_ subset, a higher proportion demonstrated dual or single expression of PD-1 and Tim-3, parallel with the notion that exhausted CD8^+^ T cells are linked to intermediate differentiation.^29^ The T_Int_ subset represents a memory sub-population in early differentiation with strong proliferation.^30^ Consistent with this, the majority of the expanded population of T cells in decidua is Tim-3^+^PD-1^+^. Given that Tim-3 signal activation could induce cell apoptosis,^31^ it was unexpected to find that these Tim-3-expressing CD8^+^ T cells displayed high Ki-67 expression but not apoptosis. Several studies have demonstrated that the function of Tim-3 varies depending on the circumstance, and that Tim-3 can also promote T-cell responses.^32, 33^ Therefore, we hypothesize that during pregnancy, Tim-3 engagement induces a distinct signaling pathway that is different from the pathway mediating cell death in CD8^+^ T cells during chronic infection. However, whether it is the maternal–fetal microenvironment that shapes this distinct signaling pathway, and what differentiates CD8^+^ T cells in this specific microenvironment, remain to be determined. Additional phenotypic profiling demonstrated that PD-1^+^Tim-3^+^CD8^+^ T cells expressed greater levels of activation and memory markers than did PD-1^−^Tim-3^−^CD8^+^ T cells, similar to the pattern observed during hepatitis C virus infection.^16^ Our study indicates that PD-1 and Tim-3 co-expression represents a T_Int_ phenotypic signature, and that these molecules may favor the survival and maintenance of this special CD8^+^ T-cell subset at the maternal–fetal interface.

Th2 bias at the maternal–fetal interface is crucial for pregnancy maintenance.^2, 6^ We demonstrated that PD-1 and Tim-3 dual positive dCD8^+^ T cells exhibited higher expression of Th2-type cytokines with stronger proliferative activity than single positive dCD8^+^ T cells while PD-1 and Tim-3 double negative CD8^+^ T cells expressed the lowest level of Th2-type cytokines. However, it appears that the Tim-3/PD-1 signaling pathway has no effect on the production of Th1-type cytokines and CD107a by dCD8^+^ T cells. This result contradicts the general opinion on the regulation of Tim-3/PD-1 signaling in Th1 cytokine production and cytolytic activity.^15, 28, 34^ Individual targeting of the Tim-3 or PD-1 pathway has variable effects on the function of dCD8^+^ T cells, whereas combined targeting of these pathways is highly effective in enhancing cytotoxicity while reducing dCD8^+^ T-cell proliferation and Th2-type cytokine production. Notably, IL-10 expression is much more enriched in the Tim-3^+^ PD-1^+^ fraction than IL-4 (Figure 3b). Together with the fact that other T-cell subsets can also produce IL-10,^35, 36^ it is possible that the Tim3^+^PD1^+^ fraction may represent a T-cell subset with a regulatory phenotype. However, TGF-*β* production by different CD8^+^ T-cell subsets did not vary with Tim-3 and PD-1 expression and was not affected by antibody treatment. Thus, we postulate that Th2-type cytokines are preferentially produced by the Tim-3^+^PD-1^+^ fraction. Given the notion that the Th2 pre-dominant pattern during early pregnancy is mostly based on CD4^+^ T cells, our data provide the evidence that Tim-3^+^PD-1^+^ dCD8^+^ T cells also have important roles in Th2 bias during pregnancy.

We also found that pregnant CBA/J females treated with Tim-3 and/or PD-1 blocking antibodies became more susceptible to fetal loss. The fetal loss was associated with dysfunction of CD8^+^ T cells by decreased GATA-3 expression, and IL-4 and IL-10 production. This dysfunction was enhanced when both Tim-3 and PD-1 were blocked. Consistent with our *in vitro* data, Th1-type cytokine production by CD8^+^ T cells was largely unaffected after injection of anti-Tim-3 and anti-PD-1 antibodies. These results suggested the differential sensitivity of Th1 and Th2 immune responses to Tim-3 and PD-1 blockade, which was quite different from what were observed in chronic disease and tumor, suggesting the development of a special immunological state during pregnancy.

We also demonstrated the differential expression of Tim-3 and PD-1 at the maternal–fetal interface and in peripheral blood between normal pregnancy and miscarriage. The number of CD8^+^ T cells co-expressing Tim-3 and PD-1 was higher in normal pregnancy. More importantly, the production of Th2-type cytokines from these cells was much higher while Th1 cytokines from these cells were lower in normal pregnancy than in miscarriage. In miscarriage, although the frequency was stable, decidual Tim-3^−^PD-1^−^CD8^+^ T cells exhibited higher Ki-67 expression, while Tim-3^+^PD-1^+^CD8^+^ T cell expansion was limited. In addition, decidual Tim-3^−^PD-1^−^CD8^+^ T cells from miscarriage produced more TNF-*α*. These data suggest that the Tim-3 and PD-1 pathways are associated with dCD8^+^ T-cell function and pregnancy outcome. The higher expression of Tim-3 and PD-1 might be a physiological response to the maternal–fetal interface environment. Co-expression of Tim-3 and PD-1 on CD8^+^ T cells could be important for the preventing tissue damage by decidua-infiltrating lymphocytes.

In summary, we described the characteristic Tim-3 and PD-1 expression patterns on dCD8^+^ T cells in both normal pregnancy and miscarriage, and elucidated their important roles in regulating dCD8^+^ T-cell function and maternal–fetal tolerance. Our data also demonstrate that, apart from Th2 bias by CD4^+^ T cells, Tim-3^+^PD-1^+^CD8^+^ T cells could also have an active role in shaping Th2 bias and regulating maternal–fetal tolerance. Moreover, approaches to promote the Tim-3/PD-1 pathway, such as treatment with agonistic Tim-3/PD-1 antibodies or galectin-9/PD-L1, may represent novel therapeutic strategies to prevent pregnancy loss. Our future studies will investigate whether the population of Tim-3^+^PD-1^+^ CD8^+^ T cell is the key mediator of tolerance in normal pregnancy by using conditioned knockout mouse models.

## Materials and Methods

### Human samples

The collection and use of the samples were approved by the Human Research Ethics Committee of the Obstetrics and Gynecology Hospital, Fudan University. Every participant signed a written informed consent form. First-trimester human villous and decidual tissues and whole peripheral blood were obtained from clinically normal pregnancies (terminated for non-medical reasons, *N*=78) and miscarriages (diagnosed as recurrent spontaneous abortion and excluding those resulting from endocrine, anatomic, genetic abnormalities, infection, etc., *N*=36). Samples were immediately collected for the isolation of peripheral blood mononuclear cells (PBMCs), DICs, and trophoblasts.

### Human cell isolation

PBMCs were isolated from peripheral blood samples of normal pregnancies and miscarriages using Ficoll density gradient centrifugation (Huajing, Shanghai).

DICs and DSCs were obtained from the decidual tissue of normal pregnancies and miscarriages. The decidual tissue was cut and digested in RPMI-1640 supplemented with collagenase type IV (1.0 mg/ml, CLS-1, Worthington Biomedical, Lakewood, NJ, USA) and DNase I (150 U/ml, Applichem, Darmstadt, Germany) as previously described.^17^ CD8^+^ T cells were isolated by magnetic affinity cell sorting using CD8 microbeads (Miltenyi Biotec, Auburn, CA, USA).

Trophoblasts were isolated by trypsin-DNase I digestion and discontinuous Percoll gradient centrifugation as described previously.^17^

### Co-culture of trophoblasts and immune cells

Freshly isolated trophoblasts were seeded at a density of 2 × 10^5^ cells/ml per well in Matrigel-coated 24-well plates overnight. The supernatants were aspirated completely, and the cells were washed twice with phosphate-buffered saline (PBS). Equal numbers of DICs or PBMCs were added to each well. In some wells, neutralizing antibodies against human HLA-C (10 *μ*g/ml, clone W6/32; BioLegend, San Diego, CA, USA), HLA-G (10 *μ*g/ml, clone 87G; BioLegend), CD80 (10 *μ*g/ml, clone 2D10; BioLegend), CD86 (10 *μ*g/ml, clone IT2.2; BioLegend), IL-4 (10 *μ*g/ml, clone 8D4-8; BioLegend), IL-10 (10 *μ*g/ml, clone JES3-9D7; BioLegend), PD-L1 (10 *μ*g/ml, clone 29E.2A3; BioLegend), or Galectin-9 (10 *μ*g/ml, clone 9M1-3; BioLegend) were added. DICs and PBMCs were also cultured with plate-bound anti-CD3 antibody (OKT-3; 5 *μ*g/ml) plus soluble anti-CD28 antibody (28.2; 1 *μ*g/ml), HTR8/SVneo cells (a human first-trimester extravillous placental trophoblast cell line) and DSCs for 48 h. In some wells, DICs and PBMCs (2 × 10^5^ cells) were plated in the upper chamber (0.4 mm pore size cell culture inserts, Millipore, Billerica, MA, USA), while trophoblasts were plated in the low chamber to establish indirect cell contact. For intracellular cytokine analysis, brefeldin A (10 *μ*g/ml, BioLegend), phorbol 12-myrstate 13-acetate (PMA) (50 ng/ml), and ionomycin (1 *μ*g/ml) were added 4 h before the end of the 48-h culture. The cells were then harvested, stained, and analyzed by flow cytometry.

### Tim-3 and PD-1 targeting experiments

Decidual immune CD8^+^ T cells were cultured (5 × 10^5^ per well) in the presence of antibodies against Tim-3 (10 *μ*g/ml, clone F38-2E2, BioLegend), PD-1 (10 *μ*g/ml, clone EH12.2H7, BioLegend), both Tim-3 and PD-1, or isotype control. After 48 h, the culture supernatant was collected and analyzed by flow cytometry or cytotoxicity assay.

### CD8^+^ T-cell cytotoxicity assay

CD8^+^ T cell-mediated cytotoxicity activity was determined by using the CytoTox 96 Non-Radioactive Cytotoxicity Assay kit (G1780; Promega, Madison, WI, USA) following the manufacturer's instructions. Decidual CD8^+^ T cells (effector cells; 100 *μ*l) at concentrations of 2.0 × 10^6^, 1.0 × 10^6^, 0.50 × 10^6^, and 0.2 × 10^6^ cells/ml and were mixed with 100 *μ*l Tros (target cells) at a concentration of 2.0 × 10^4^ cells/ml, resulting in effector : target (E : T) ratios of 100 : 1, 50 : 1, 25 : 1, and 10 : 1, respectively. Then the effector:target ratio of 100 : 1 was chosen in the later cytotoxicity assay. The dCD8^+^ T cells were separately treated with Tim-3 and/or PD-1 antibody or varying concentrations of recombinant human IL-4 (Peprotech, Rocky Hill, NJ, USA) and IL-10 (Peprotech). Alternatively, the cells were sequentially stimulated with anti-Tim-3 and/or PD-1 antibody plus IL-4 and IL-10 for 48 h before mixture with Tros at a ratio of 100 : 1. Each E : T ratio was examined in triplicate.

### CD8^+^ T-cell viability assay

We used the Cell Counting Kit-8 (CCK-8; Dojindo, Kumamoto, Japan) to evaluate cell viability. CD8^+^ T cells (1 × 10^5^/well) were cultured in 96-well plates and treated for 48 h with Tim-3 and/or PD-1 antibody as described earlier. Thereafter, 10 *μ*l CCK-8 was added into each well and incubated at 37 °C for 0.5–4 h. The optical density at 450 nm was determined using an enzyme-linked immunosorbent assay (ELISA) Reader (Molecular Devices, Sunnyvale, CA, USA) to measure cell viability.

### Mice

CBA/J female and BALB/c male mice were purchased from Huafukang (Beijing, China) and maintained in an animal facility according to institutional and National Institutes of Health guidelines. Eight-week-old females were mated to BALB/c males to induce pregnancy and inspected every morning for vaginal plugs. The day of visualization of a plug was designated as day 0.5 of pregnancy. Pregnant mice were monitored at day 10.5 of pregnancy to assess the effect of anti-Tim-3 and anti-PD-1 antibody treatment on pregnancy. Live fetus per uterus=the number of all fetuses per uterus–the number of resorbed embryos.

### Mouse treatment protocol

Pregnant females received injections of anti-Tim-3 antibody (clone RMT3-23, BioLegend), anti-PD-1 antibody (clone RMP1-14, BioLegend), both antibodies, or PBS i.p. at doses of 500, 250, and 250 mg on days 4.5, 6.5, and 8.5, respectively. This protocol was based on the previous publications.^37, 38^

### Preparation of mouse cells

Uteri from pregnant mice were dissected free from the mesometrium and removed by cuts at the ovaries and cervix. The fetal and placental tissues were carefully removed. Minced uteri were digested in RPMI-1640 supplemented with collagenase type IV and DNase I for 30 min at 37 °C with gentle agitation. The spleen was aseptically excised and stored in RPMI-1640. A single-cell suspension was made by using a 10-ml syringe plunger to pass spleen tissue into fresh wash medium through a 70-*μ*m mesh strainer. Cells were cultured in RPMI-1640 supplemented with 10% FBS, 100 U/ml penicillin, 100 *μ*g/ml streptomycin, and 1 *μ*g/ml amphotericin B at 37 °C in 5% CO_2_.

### Flow cytometry

Cell surface molecular expression and intracellar cytokine production were evaluated using flow cytometry. FITC-conjugated anti-human or anti-mouse CD8, PE-conjugated anti-human Tim-3, or anti-mouse T-bet or GATA-3, PE/CY7-conjugated anti-human CD45RA or IL-10 or TNF-*α*, or IFN-*γ* or transforming growth factor (TGF)-*β*, PerCP/Cy5.5-conjugated anti-human CCR7, APC-conjugated anti-human PD-1 or IL-5, or anti-mouse TNF-*α* or IL-10, Brilliant Violet 421-conjugated anti-human CD27 or CD107a or Ki67 or IL-4, or anti-mouse IFN-*γ* or IL-4 (Biolegend) antibodies were used. For intracellular staining, cells were fixed and permeabilized using the Fix/Perm kit (Biolegend). Flow cytometry was performed on a Beckman-Coulter CyAN ADP cytometer and analyzed with FlowJo software (Tree Star, Ashland, OR, USA).

### Statistical analysis

The statistical significance of differences between two groups was determined by the *post-hoc* Dunnett *t*-test. Multiple groups were analyzed with GraphPad Prism Version 5 (GraphPad Software, La Jolla, CA, USA) by one-way or two-way ANOVA with Bonferroni post *t*-tests. For all statistical tests, *P*-values <0.05 were considered as statistically significant.

## Abbreviations

Tim-3: T-cell immunoglobulin mucin-3
PD-1: programmed cell death-1
DICs: decidual immune cells
Tros: trophoblasts
PBMCs: peripheral blood mononuclear cells
EVT: extravillous trophoblast
dCD8^+^ T: decidual CD8^+^ T
pCD8^+^ T: peripheral CD8^+^ T
TNF-*α*: tumor necrosis factor-*α*
IFN-*γ*: interferon-*γ*
IL-10: interleukin-10
TGF-*β*: transforming growth factor-*β*
PMA: phorbol 12-myrstate 13-acetate
PBS: phosphate-buffered saline
MHC: major histocompatibility complex
