# Supplementary material for: PD-1 and Tim-3 pathways are associated with regulatory CD8+ T-cell function in decidua and maintenance of normal pregnancy
Source: Cell Death Dis. 2015 May 7;6(5):e1738–. doi: 10.1038/cddis.2015.112 (PMC4669692; doi:10.1038/cddis.2015.112)
Supplement: Supplementary Figure Legends [file cddis2015112x1.docx]

**Figure S1.** The expression of PD-1 and Tim-3 on CD8^+^ T cells from decidua and peripheral blood during human early pregnancy.

**Figure S2.** Proliferation of peripheral CD8^+^ T cells during human early pregnancy.

**Figure S3.** The expression of CD107a and TGF-β1 in decidual CD8^+^ T cells during human early pregnancy.

**Figure S4.** The cytotoxic activity of decidual CD8^+^T cells towards human trophoblast cells.

**Figure S5.** The effect of targeting Tim-3 and PD-1 signaling pathways on decidual CD8^+^ T cell biological function.

**Figure S6.** The effect of Tim-3 and PD-1 blockade on cytokine and transcription factor expression in murine spleen CD8^+^ T cells during early pregnancy.
