# Supplementary figures and images for: PD-1 and Tim-3 pathways are associated with regulatory CD8+ T-cell function in decidua and maintenance of normal pregnancy
Source: Cell Death Dis. 2015 May 7;6(5):e1738–. doi: 10.1038/cddis.2015.112 (PMC4669692; doi:10.1038/cddis.2015.112)

Figure S1

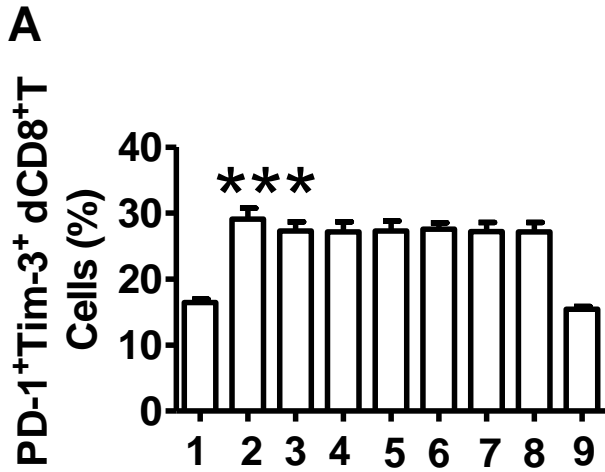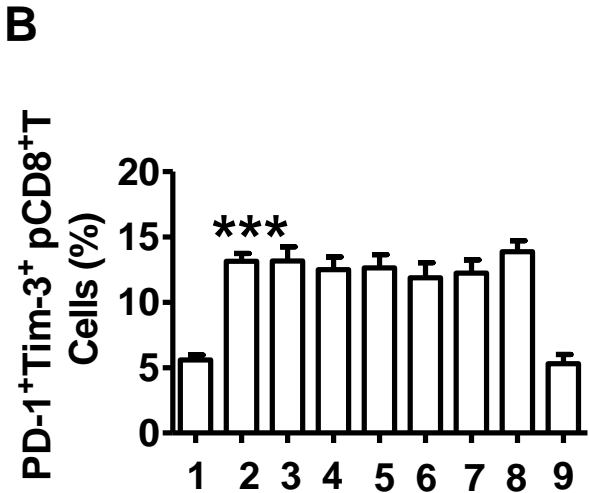

Figure S2

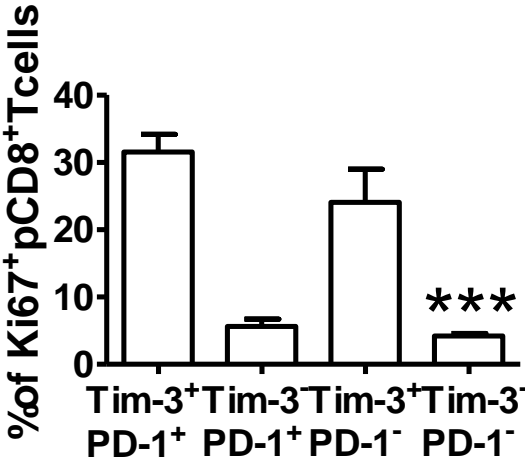

Figure S3

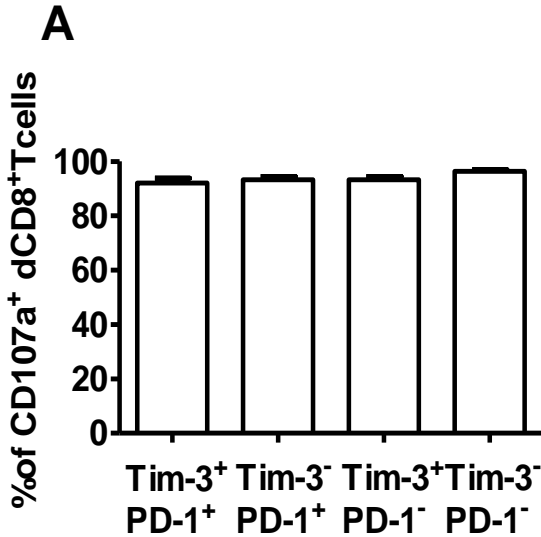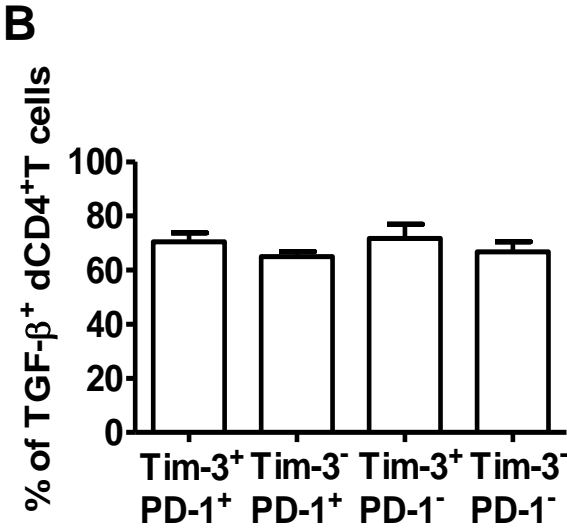

Figure S4

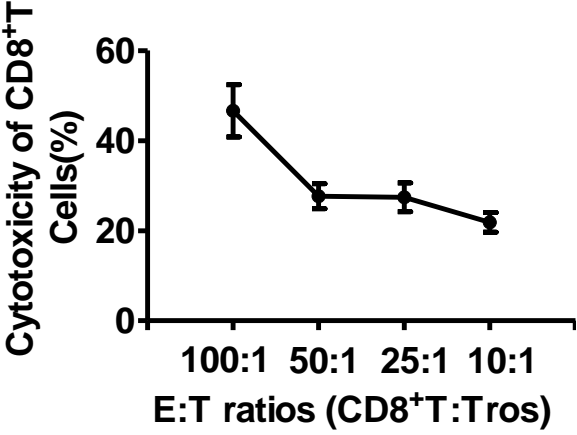

Figure S5

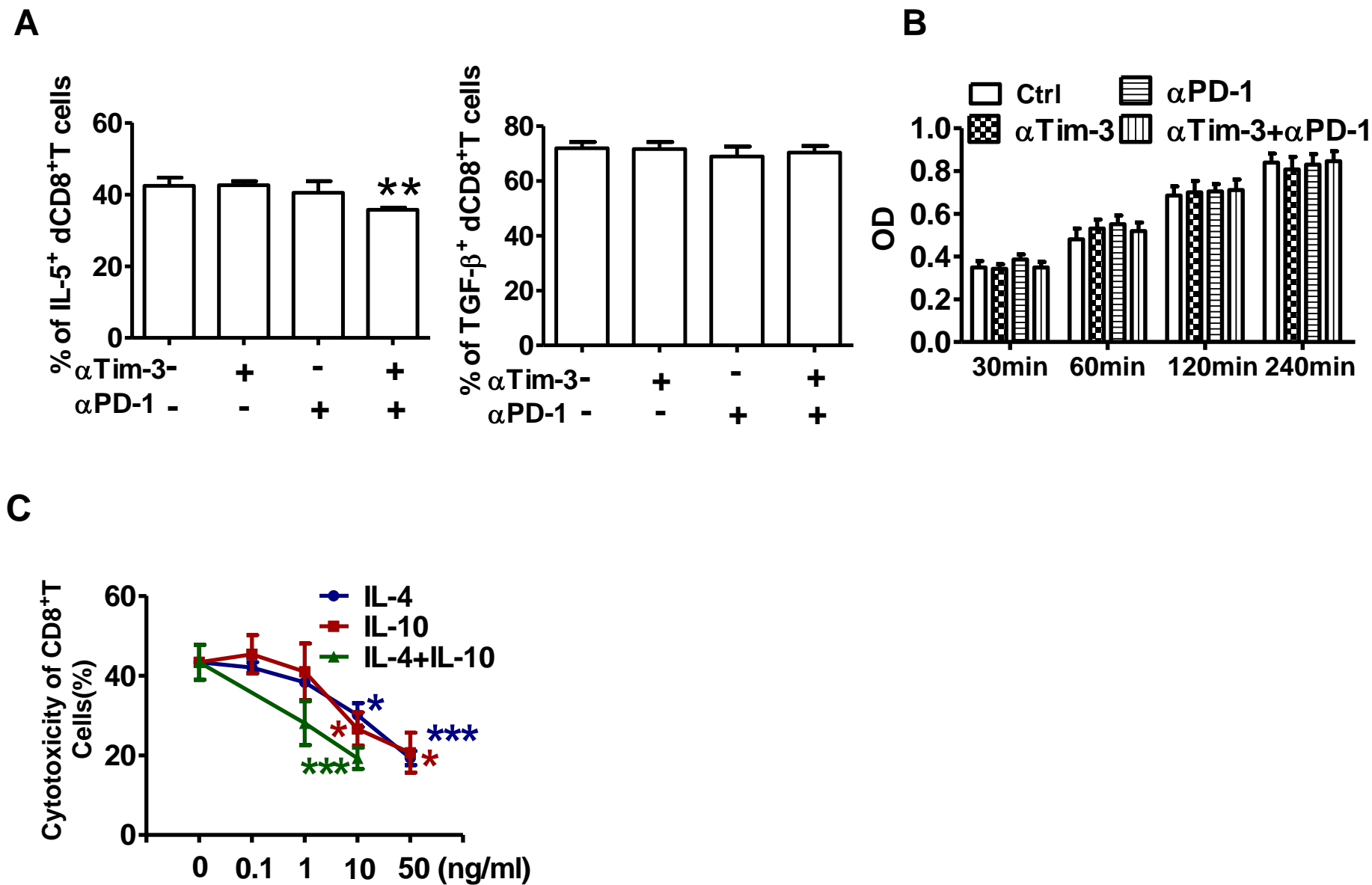

Figure S6

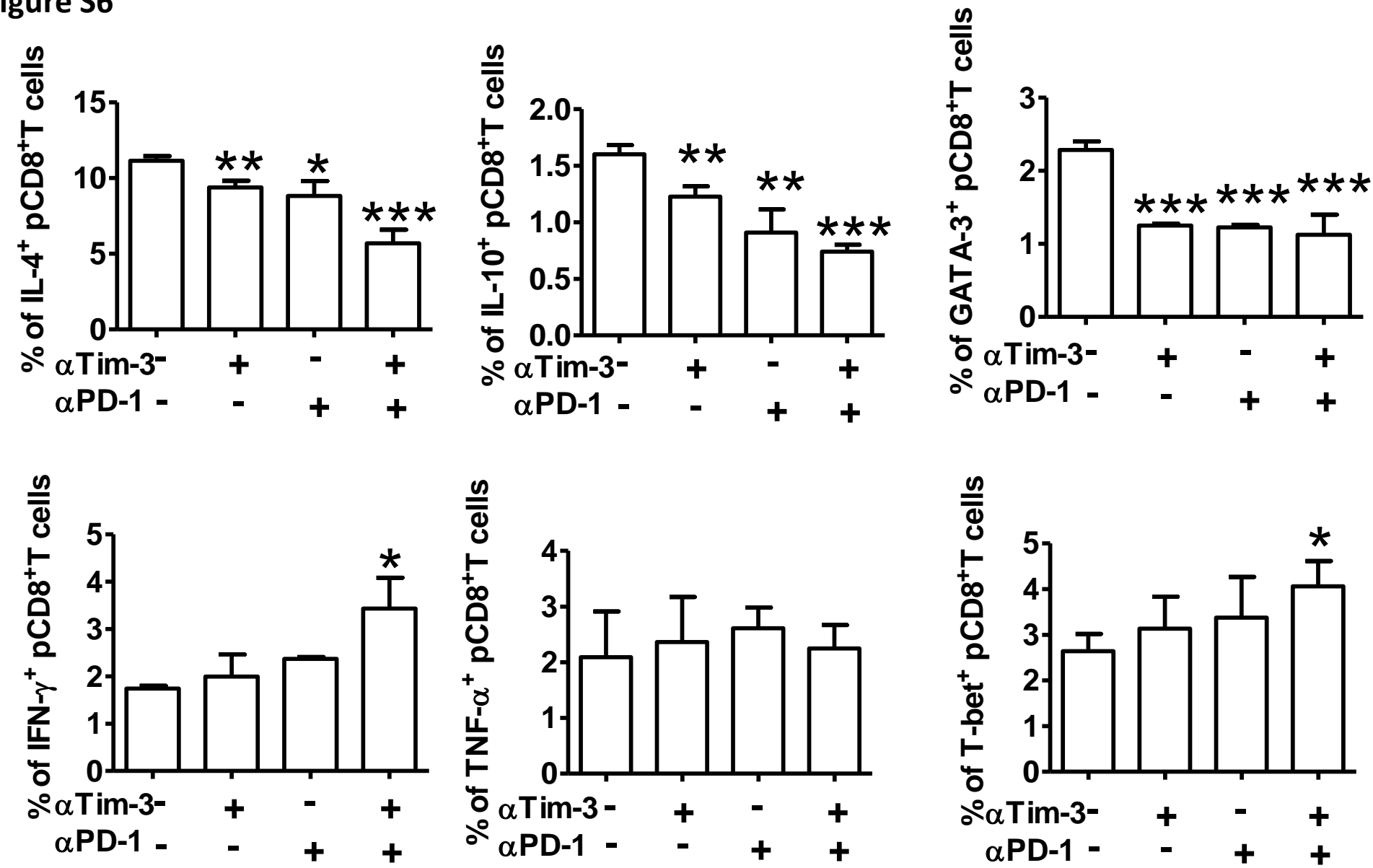

Supplement: Supplementary Figures [file cddis2015112x2.pdf]
